# Hesperidin Effects on Gut Microbiota and Gut-Associated Lymphoid Tissue in Healthy Rats

**DOI:** 10.3390/nu11020324

**Published:** 2019-02-02

**Authors:** Sheila Estruel-Amades, Malén Massot-Cladera, Francisco J. Pérez-Cano, Àngels Franch, Margarida Castell, Mariona Camps-Bossacoma

**Affiliations:** Secció de Fisiologia, Departament de Bioquímica i Fisiologia, Facultat de Farmàcia i Ciències de l’Alimentació, Universitat de Barcelona (UB); Institut de Recerca en Nutrició i Seguretat Alimentària (INSA-UB), UB, 08007 Barcelona, Spain; sheilaestruel@ub.edu (S.E.-A.); malen.massot@ub.edu (M.M.-C.); franciscoperez@ub.edu (F.J.P.-C.); angelsfranch@ub.edu (À.F.); marionacamps@ub.edu (M.C.-B.)

**Keywords:** flavanone, flavonoids, immunoglobulin A, intestinal immunity, prebiotic, polyphenol

## Abstract

Hesperidin, found in citrus fruits, has shown a wide range of biological properties. Nonetheless, a more in-depth investigation is required on the effects on the immune system, and in particular, on the gut-associated lymphoid tissue, together with its relationship with the gut microbiota. Therefore, we aimed to establish the influence of oral hesperidin administration on the intestinal lymphoid tissue and on the gut microbiota composition in healthy animals. Lewis rats were orally administrated 100 or 200 mg/kg hesperidin three times per week for four weeks. Microbiota composition and IgA-coated bacteria were determined in caecal content. Mesenteric lymph node lymphocyte (MLNL) composition and functionality were assessed. IgA, cytokines, and gene expression in the small intestine were quantified. Hesperidin administration resulted in a higher number of bacteria and IgA-coated bacteria, with changes in microbiota composition such as higher *Lactobacillus* proportion. Hesperidin was also able to increase the small intestine IgA content. These changes in the small intestine were accompanied by a decrease in interferon-γ and monocyte chemotactic protein-1 concentration. In addition, hesperidin increased the relative proportion of TCRαβ+ lymphocytes in MLNL. These results show the immunomodulatory actions of hesperidin on the gut-associated lymphoid tissue and reinforce its role as a prebiotic.

## 1. Introduction

Polyphenols, extensively found in plants as a product of their secondary metabolism [1] can be classified into different groups regarding their chemical structure: Phenolic acids, flavonoids, anthocyanidins, stilbenes, and lignans [2]. Within the flavonoid family, the most distinguished subgroups are flavonols (e.g., quercetin, kaempferol, and myricetin), flavanones (e.g., eriodictyol, hesperetin, and naringenin), isoflavones (e.g., daidzein, genistein, and glycetein), flavones (e.g., apigenin, and luteolin), flavan-3-ols (e.g., catechin), and anthocyanins (e.g., cyanidin, delphinidin, and malvidin) [3]. The biological activity of flavonoids in human or animal health and their protective role in several diseases have been widely described [4,5]. There is a growing interest in flavonoids for their anti-inflammatory [6] and anti-diabetic [7] properties, as well as their microbial modulatory actions [8].

Hesperidin is the major flavanone present in citrus fruits, such as orange [9,10]. It is composed by hesperetin being conjugated to rutinose. After hesperidin intake, in the small intestine this flavanone is poorly absorbed via the paracellular pathway and it is highly dependent on the conversion to hesperetin [5,11]. Hesperidin reaches the large intestine where gut microbiota cleaves the attached rutinose moiety, forming hesperetin for further colonic absorption [12]. Hesperidin is gaining attention due to its different biological activities [3]. In this context, it has been considered a potential protective factor in neurodegenerative diseases [13], by reducing neuro-inflammation in experimental stroke [14]. Moreover, its anti-oxidant [15,16], anti-depressive [17], anti-cancer [18], and immunomodulatory properties have also been described [19,20,21,22,23]. In particular, hesperidin has been shown to increase the production of anti-inflammatory cytokines in vitro [19], to exert an anti-asthmatic effect [20,21], to alter the CD4/CD8+ T cell ratio in the intestine wall of mice infected with *Aeromonas hydrophila* [22], and to influence the lymphocyte composition and functionality of the gut-associated lymphoid tissue in immunized rats [23]. Overall, the immunomodulatory properties of hesperidin were observed in vitro or in infection/immunization models in which the immune system was triggered. Nevertheless, no studies have shown the immune effects of this flavanone in health status. 

On the other hand, as far as we know, Unno et al. [24] in the only existing study on the influence of citrus flavanones on the gut microbiota, included them in rat food and showed the prebiotic-like effects of a hesperetin-enriched diet, but not a diet containing hesperidin. In this context, the relationship between the gut microbiota and the function of the gut-associated lymphoid tissue must be emphasized, as its close interaction are well established [25]. Indeed, the intestinal mucosa may be considered as an immunological niche as it hosts a complex immune-functional organ comprised of immunocompetent cells, their products, such as secretory IgA, and the microbiota [25].

While some studies have focused on the influence of hesperidin on the immune response, an in-depth investigation is needed into the effects of hesperidin on the gut-associated lymphoid tissue, which hesperidin reaches first, and moreover, where it can interact with gut microbiota contributing to the crosstalk between gut bacteria and intestinal immune tissue. Therefore, the aim of the present study was to establish the influence of oral hesperidin administration on the function of the gut-associated lymphoid tissue, including the mesenteric lymph node lymphocyte phenotype characterization, and on the microbiota composition in healthy rats. In fact, even in good health, intestinal immune tissue is continuously active, distinguishing innocuous antigens (from food and gut microbiota) from pathogenic microorganisms [26]. The dosage used in the current intervention (100 and 200 mg/kg body weight by oral gavage, three times per week for four weeks) had already been used in a previous study, producing higher immunomodulatory effects than the incorporation of the hesperidin in the rat food [22].

## 2. Materials and Methods 

### 2.1. Animals and Diets

The experimental procedure of this study was approved by the Ethical Committee for Animal Experimentation of the University of Barcelona and the Catalonia Government (CEEA/UB Ref. 464/16 and DAAM 9257, respectively). 

Three-week-old male Lewis rats (*n* = 18) were purchased from Janvier Labs (Saint-Berthevin Cedex, France) and housed in polycarbonate cages (3 animals per cage) with large fibrous-particle bedding and tissue papers as enrichment, and monitored daily in a controlled environment of temperature and humidity, in a 12/12 h light/dark cycle in the Faculty of Pharmacy and Food Science animal facility. Water and food (Teklad Global 14% Protein Rodent Maintenance Diet, Teklad, Madison, WI, USA, Supplementary Table S1) were provided ad libitum throughout the study. Body weight (BW) was monitored during the study, as well as the food and water consumption in each cage. Animals were randomly assigned into three groups (six animals/group): Reference (REF), H100, and H200 groups. The H100 group received 100 mg/kg BW hesperidin, the H200 group received 200 mg/kg BW hesperidin, and the REF group received the same volume of 0.5% carboxymethylcellulose that was used as a vehicle (1 mL/100 g BW). Hesperidin was given by oral gavage three times a week for four weeks. The hesperidin, kindly provided by Ferrer HealthTech (Murcia, Spain), had a purity of 95.5%, containing 2% isonaringine, 1.5% didimine, and other impurities, as determined by high-performance liquid chromatography.

### 2.2. Sample Collection and Processing

Blood and feces were collected weekly throughout the study. Serum was kept at −20 °C until immunoglobulin quantification. Fecal samples were dried overnight at 37 °C and weighed in order to obtain the fecal homogenates (20 mg/mL), as previously described [23].

After four weeks, animals were anaesthetized intramuscularly with ketamine (Merial Laboratories S.A. Barcelona, Spain) and xylazine (Bayer A.G., Leverkusen, Germany) (90 mg/kg and 10 mg/kg, respectively). In addition to blood and feces, urine (directly from urine bladder) and caecal samples were collected. Moreover, small intestine and mesenteric lymph node (MLN) samples were obtained.

Caecal content was weighed and homogenized to establish microbiota composition as well as to determine IgA content. For microbiota analysis, a part of the caecal homogenates was fixed overnight using 4% paraformaldehyde (Merck, Madrid, Spain). After a low centrifugation, pellets were resuspended with 1:1 phosphate buffered saline (PBS):ethanol, stored at −20 °C for at least 1 h, and maintained at −80 °C until analysis. For caecal IgA, the rest of the caecal homogenate was centrifuged (538 g, 5 min, 4 °C) and supernatants were kept at −80 °C until analysis.

To obtain small intestine washes, the distal small intestine was obtained and processed as previously described [27]. Moreover, a middle part of the small intestine was kept in RNAlater^®^ (Ambion, Life Technologies, Austin, TX, USA) until the determination of gene expression of some molecules by real-time polymerase chain reaction (RT-PCR). 

### 2.3. Lymphocyte Isolation from Mesenteric Lymph Nodes

Lymphocytes from MLNs (MLNL) were isolated, as previously detailed [28]. Briefly, MLNs were obtained in aseptic conditions and immediately immersed in Roswell Park Memorial Institute (RPMI) medium supplemented with 10% heat-inactivated fetal bovine serum (FBS), 100 IU/mL streptomycin-penicillin, 2 mM L glutamine and 0.05 mM 2-mercaptoethanol (Sigma-Aldrich, Madrid, Spain). MLNs were smashed in a sterile mesh cell strainer (40 µm, Thermo Fisher Scientific, Barcelona, Spain). After centrifugation, the recovered pellet was resuspended with Dulbecco’s Modified Eagle Medium (DMEM-GlutaMAX, Gibco™, Thermo Fisher Scientific). Cell counting and viability were assessed by a Countess™ automated cell counter (Invitrogen™, Thermo Fisher Scientific, Barcelona, Spain). 

### 2.4. Quantification of Urine Total Phenolic Content

Total phenolic content in urine was determined according to Folin–Ciocalteu’s method using a gallic acid standard curve (0–32 µg/mL), as previously described [28]. 

### 2.5. Fluorescence In Situ Hybridization of Gut Microbiota

The bacterial groups present in caecal content were characterized by means of the fluorescent in situ hybridization (FISH) technique coupled to flow cytometry (FCM) analysis using group- or genus-specific fluorochrome-conjugated probes (Table 1), which target the bacterial 16S rRNA (Sigma-Aldrich, Madrid, Spain), as previously established [29,30,31]. FCM analysis (FacsAria SORP sorter, BD, San José, CA, USA) was carried out in the flow cytometry unit of the Scientific and Technological Centres of the University of Barcelona (CCiT-UB). Analysis was performed using FlowJo v10 software (Tree Star, Inc., Ashland, OR, USA). Microbiota data were expressed as total bacteria counts/g caecal content, as well as the relative percentage of each bacterial group (the sum of all percentages of each studied probe was considered as 100%).

### 2.6. Quantification of Bacteria Coated to IgA

IgA-coated bacteria in caecal content were determined, as previously described [31]. After centrifuging the diluted caecal homogenates in 1% (*v*/*v*) FBS/PBS, the resulting pellet was resuspended in a dilution of fluorescein isothiocyanate (FITC)-anti-rat Ig antibodies (Abcam, Cambridge, UK) in 1% FBS/PBS and incubated for 30 min at 4 °C in the dark. Labelled samples were mixed with propidium iodide (1 mg/mL, Sigma–Aldrich) 15 min prior to FCM analysis in order to label total bacteria. The counts of IgA-coated bacteria were established from the IgA-coated bacteria proportion and the counts of bacteria of each sample using Commercial Flow CheckTM Fluorospheres (Beckman Coulter, Inc., Hialeah, FL, USA) combined with propidium iodide. IgA-coated bacteria results were expressed both as caecal IgA+ bacteria counts/g caecal content and as the relative percentage of IgA+ bacteria.

### 2.7. MLN Lymphocyte Composition

MLNLs (500,000 cells/tube) were stained using mouse anti-rat monoclonal antibodies conjugated to FITC, phycoerythrin (PE), peridinin-chlorophyll-protein (PerCP), allophycocyanin (APC) or APC-cyanine (Cy)7. The antibodies used herein were anti-TCRαβ, anti-CD8α, anti-CD4, anti-TCRγδ and anti-CD45RA (BD Biosciences, San Diego, CA, USA). Cells were mixed with PBS containing 2% FBS and 1% NaN_3_ and stained, as previously described [28]. The data were acquired with a Gallios™ Cytometer (Beckman Coulter, Miami, FL, USA) in the CCiT-UB and assessed by the Flowjo v10 software (Tree Star, Inc., Ashland, OR, USA). Results are expressed as percentages of positive cells in the lymphocyte population, selected according to their forward-scatter characteristics (FSC) and side-scatter characteristics (SSC).

### 2.8. MLN Lymphocyte Proliferative Capacity

T-lymphocyte activation was carried out in 96-well plates previously coated with anti-CD3/CD28, as previously described [39]. MLNLs (10^5^ cells/well) were incubated in quadruplicate with or without stimulus. After 48 h (37 °C, 5% CO_2_), lymphocytes were incubated with the pyrimidine analogue bromodeoxyuridine (BrdU) in order to determine the proliferative capacity using the BrdU Cell Proliferation Assay kit (Merck Millipore, Darmstadt, Germany) following the manufacturer’s instructions.

### 2.9. Cytokine Quantification

Concentrations of interleukin (IL)-4, IL-10, interferon (IFN)-γ, tumor necrosis factor (TNF)-α and monocyte chemotactic protein (MCP)-1 were quantified in MLNL supernatants after 48 h of anti-CD3/CD28 monoclonal antibody stimulation, and in small intestine washes by ProcartaPlex^®^ Multiplex Immunoassay (Affymetrix, eBioscience, San Diego, CA, USA), as detailed in previous studies [23].

### 2.10. Immunoglobulin Quantification by ELISA 

IgA concentration in small intestine washes, serum, faecal and caecal homogenates was quantified using a sandwich enzyme-linked immunosorbent assay (ELISA) technique with a rat IgA ELISA Quantification Set (A110-102) from Bethyl Laboratories (Montgomery, TX, USA). Intestinal and serum IgM and IgG concentrations were quantified using rat IgM and IgG ELISA Quantification sets (Bethyl Laboratories, A110-100 and A110-1364, respectively). The kits were applied following the manufacturer’s instructions. Absorbance was measured on a microplate photometer (Labsystems Multiskan, Helsinki, Finland) at 492 nm. Data were interpolated by means of Ascent v.2.6 software (Thermo Fisher Scientific, S.L.U, Barcelona, Spain), according to the respective standard curves.

### 2.11. Gene Expression by Real-Time Polymerase Chain Reaction (RT-PCR)

The RNA isolation from small intestine samples was done by the RNeasy^®^ mini kit (Qiagen, Madrid, Spain) after homogenization in a FastPrep^®^-24 (MP Biomedicals) for 30 s as previously detailed [40]. After RNA concentration and purity determination by a NanoDrop spectrophotometer (NanoDrop Technologies, Wilmington, DE, USA), the RNA was reverse-transcribed using random hexamers and TaqMan^®^ Reverse Transcription Reagents (Applied Biosystems, AB, Weiterstadt, Germany). The RT-PCR was performed using the ABI Prism 7900 HT quantitative RT-PCR system with the following specific PCR TaqMan^®^ primers from AB: IgA (331943, made to order); TGF β1 (Rn00572010_m1; Inventoried (I)); CCR9 (Rn00597283_m1, I); CD40 (Rn01423584_g1, I); CCL25 (Rn01403352_m1, I); and CCL28 (Rn00586715_m1, I). Quantification of the target genes was normalized with the housekeeping gene HPRT (Rn01527840_m1, I). The SDS s2.4 software (AB) was used to analyze the expression data. Relative gene expression levels are represented as a percentage compared with the REF group (considered 100% of gene expression) using the 2^−ΔΔCt^ method, as previously described [37]. Results are expressed as the mean ± standard error of the percentage of these values. 

### 2.12. Statistical Analysis

Scientific data analysis and graphing software SigmaPlot (version 12.0, San Jose, CA, USA), was used to create graphics. Statistical analysis of the data was performed using IBM Social Sciences Software Program (SPSS, version 22.0, Chicago, IL, USA). Variance equality and homogeneity of the data were assessed by Levene’s and Shapiro–Wilk tests, respectively. When the equality and normality of the results were established, a one-way ANOVA test was carried out. When significant differences were obtained, Bonferroni’s post hoc test was carried out between groups. Kruskal–Wallis (KW) test was used when results were neither equally nor normally distributed. When significant differences were obtained by the KW test, a Mann–Whitney *U* test was performed between groups. To assess differences in parameters throughout the study (e.g., body weight or serum immunoglobulins), a repeated measure ANOVA test was performed. When significant differences were obtained, a Mann–Whitney *U* test was carried out between groups. To assess the correlation between variables, a Pearson correlation was used. Significant differences were considered when *p* < 0.05.

## 3. Results

### 3.1. Influence of Hesperidin on Body Weight, Food Intake and Urine Flavonoid Content

The administration of 100 mg/kg or 200 mg/kg of hesperidin three times per week for four weeks did not modify the body weight compared to the REF group (Table 2). Similarly, no changes were observed in either food or water intake due to the hesperidin during the intervention (Table 2). 

**Figure 1:**
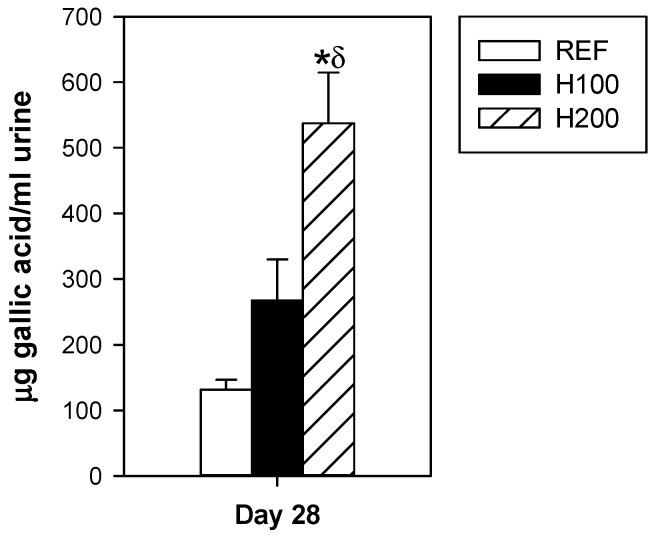
Effect of hesperidin administration on total polyphenol content in urine. Data are expressed as mean ± standard error (*n* = 5–6). Statistical difference: * *p* < 0.05 versus REF group, ^δ^ *p* < 0.05 versus H100 group (one-way ANOVA).

At the end of the study, although both hesperidin administrations resulted in a dose-dependent total polyphenol increase in urine, only the highest dose reached significance (Figure 1). These results evidence its intestinal absorption.

### 3.2. Influence of Hesperidin on Caecal Microbiota 

**Figure 2:**
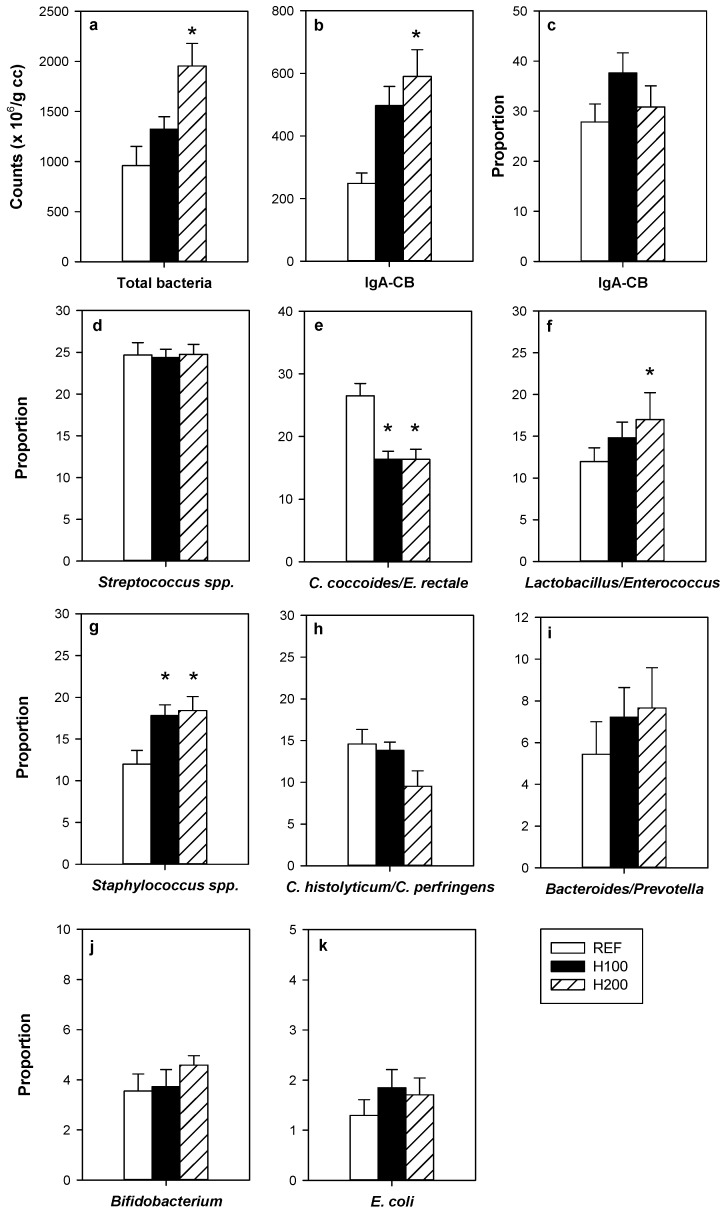
Effect of hesperidin administration on bacterial groups determined by FISH-FCM in caecal homogenates. (**a**) Counts of total bacteria; (**b**) counts of IgA-coated bacteria; (**c**) proportion of IgA-coated bacteria; proportions of (**d**) *Streptococcus* spp.; (**e**) *Clostridium coccoides/Eubacterium rectale*; (**f**) *Lactobacillus/Enterococcus*; (**g**) *Sstaphylococcus* spp.; (**h**) *Clostridium histolyticum/Clostridium perfringens*; (**i**) *Bacteroides/Prevotella*; (**j**) *Bifidobacterium*; and (**k**) *Escherichia coli*. Data are expressed as mean ± standard error (*n* = 5–6). Statistical difference: * *p* < 0.05 versus REF group (one-way ANOVA).

The administration of the highest dose of hesperidin increased, by twofold, the amount of total bacteria in the caecum compared to that found in the REF group (Figure 2a). Such effect was not observed in the H100 group. No significant correlation between caecal bacteria and urine polyphenol content was found (*r* = 0.505, *p* = 0.113). 

In addition to increasing the total amount of caecal bacteria, hesperidin administration promoted the growth of particular bacterial groups as observed by the increased proportions of *Lactobacillus/Enterococcus* (Figure 2f) and *Staphylococcus* (Figure 2g), whereas decreased that of *Clostridium coccoides/Eubacterium rectale* (Figure 2e). Moreover, when considering the bacterial counts, the highest dose of hesperidin induced increases in *Streptococcus*, *Lactobacillus/Enterococcus*, *Staphylococcus*, *Bacteroides/Prevotella*, *Bifidobacterium*, and *Escherichia coli* groups (Supplementary Figure S1). 

The number of caecal bacteria coated with IgA significantly increased after the administration of hesperidin (*p* = 0.008 H200 vs. REF group and *p* = 0.065 H100 vs. REF group). Nevertheless, the proportion of bacteria coated with IgA was similar between groups (Figure 2b,c). 

### 3.3. Influence of Hesperidin on MLNL Composition and Functionality

**Figure 3:**
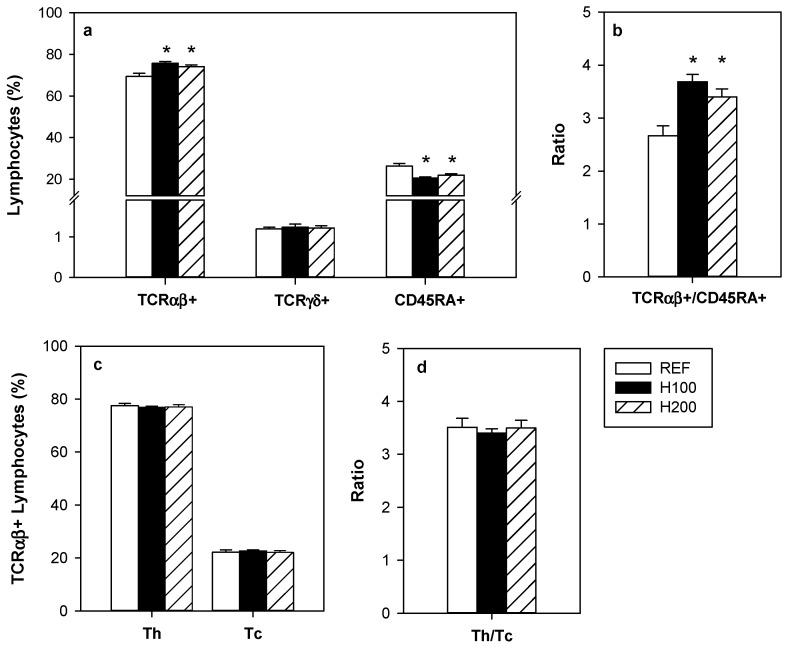
Effect of hesperidin administration on the proportion of mesenteric lymph node lymphocytes. (**a**) TCRαβ+, TCRγδ+ and CD45RA+ lymphocytes; (**b**) TCRαβ+/CD45RA+ ratio; (**c**) Th (TCRαβ+CD4+) and Tc (TCRαβ+CD8+) lymphocytes; and (**d**) Th/Tc ratio. Data are expressed as mean ± standard error (*n* = 5–6). Statistical difference: * *p* < 0.05 versus REF group (one-way ANOVA).

Hesperidin, at both doses, increased significantly the proportion of TCRαβ+ lymphocytes in the MLN without modifying that of TCRγδ+ cells (Figure 3a). Reciprocally, the proportion of B lymphocytes (CD45RA+) was decreased by the hesperidin administration, and consequently the TCRαβ+/CD45RA+ ratio increased around 33% due to 100 and 200 mg/kg hesperidin (Figure 3a,b).

Regarding the TCRαβ+ cell subsets, no significant differences were observed in either Th (TCRαβ+CD4+) or Tc (TCRαβ+CD8+) cells, indicating that the increase in TCRαβ+ cells affected both subsets similarly (Figure 3c). Accordingly, no significant differences were observed between groups in the Th/Tc ratio (Figure 3d).

**Figure 4:**
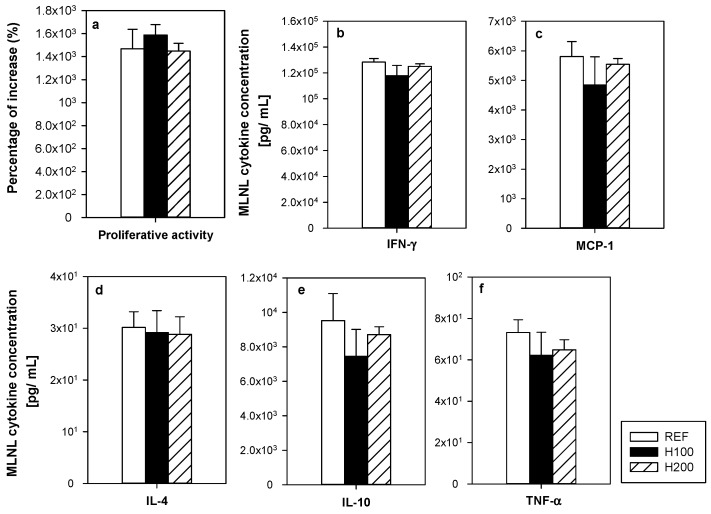
Effect of hesperidin administration on (**a**) the mesenteric lymph node lymphocyte (MLNL) proliferative activity; and (**b**–**f**) cytokine release by MLNL after anti-CD3/CD28 stimulation. Data are expressed as mean ± standard error (*n* = 5–6).

To assess the functionality of the MLNL, their proliferative capacity and the cytokine pattern released after in vitro stimulation were determined (Figure 4a–f). Oral hesperidin administration at any of the doses used did not modify the MLNL ability to proliferate as a result of anti-CD3/CD28 stimulus (Figure 4a). In addition, the hesperidin administration did not significantly alter the secretion pattern of cytokines (IFN-γ, MCP-1, IL-4, IL-10, and TNF-α) released by activated MLNL (Figure 4b–f). 

### 3.4. Effect of Hesperidin on Small Intestine Cytokines and Gene Expression

**Figure 5:**
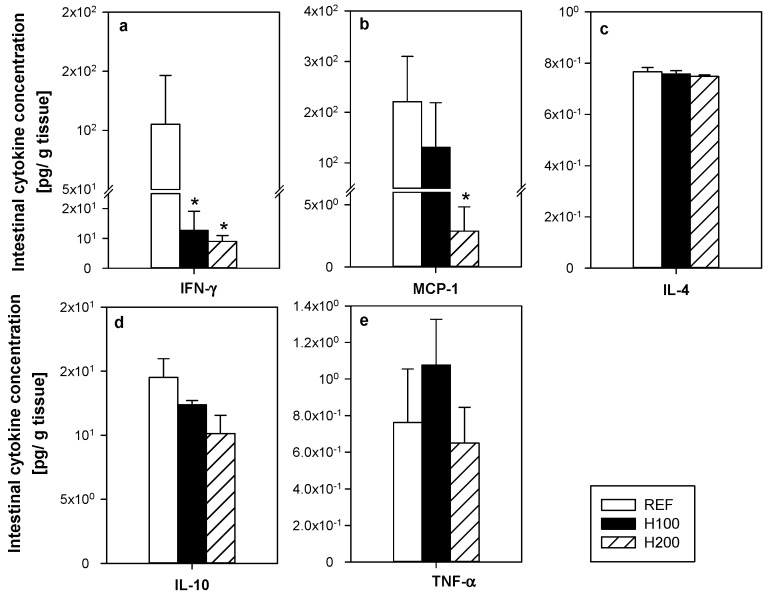
Effect of hesperidin administration on the cytokine concentration in the small intestine washes (**a**–**e**). Data are expressed as mean ± standard error (*n* = 5–6). Statistical difference: * *p* < 0.05 versus REF group (Mann–Whitney *U* test).

Hesperidin administration changed the cytokine profile in the small intestine wash (Figure 5a–e). Both doses of hesperidin significantly reduced the concentration of IFN-γ in this compartment (Figure 5a), whereas the highest dose significantly decreased that of MCP-1 (Figure 5b). The content of IL-4, IL-10, and TNF-α in the intestinal compartment was not affected by the administration of the flavanone (Figure 5c–e).

Regarding the gene expression study, hesperidin intervention did not alter the expression of the genes encoding for IgA, TGF-β, CCR9, CD40, CCL25, and CD28 in the small intestine (Table 3).

### 3.5. Effect of Hesperidin on Intestinal Immunoglobulins

**Figure 6:**
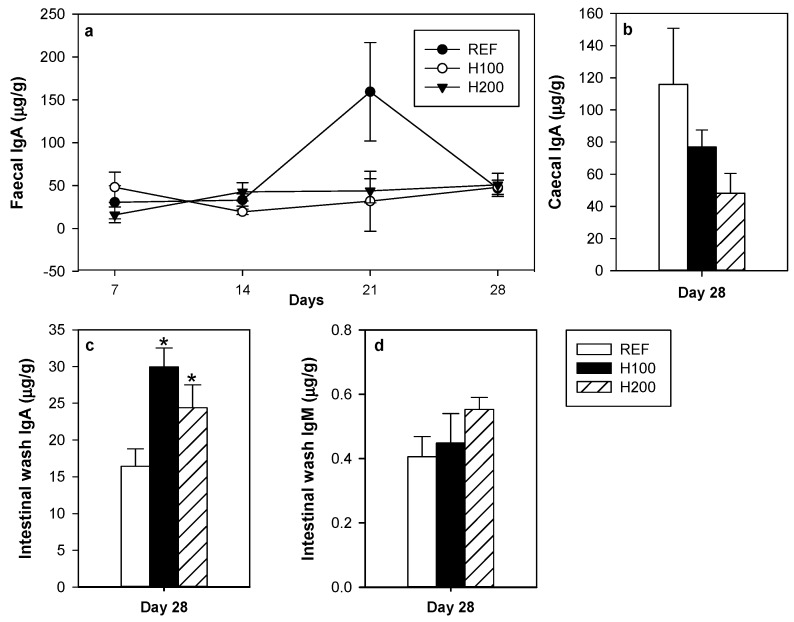
Effect of hesperidin administration on (**a**) IgA concentration in faeces collected throughout the study; (**b**) caecum content; (**c**) small intestine wash; and on (**d**) IgM concentration in the small intestine wash. Data are expressed as mean ± standard error (*n* = 5–6). Statistical difference: * *p* < 0.05 versus REF group (one-way ANOVA).

The effect of hesperidin administration on immunoglobulin content at several intestinal compartments was established (Figure 6a–d). Flavanone administration did not modify either the time-course of faecal IgA during the four weeks (Figure 6a) or the caecal IgA content at the end of the study (Figure 6b). Interestingly, both hesperidin doses resulted in a nearly twofold increase of the small intestine content of IgA (Figure 6c). There was no correlation between small intestine IgA content and the urine polyphenol concentration (*r* = 0.247, *p* = 0.491). However, a positive correlation was observed between small intestine IgA levels and T/B ratio in MLNL (*r* = 0.644, *p* = 0.013). No effect on small intestinal IgM was detected (Figure 6d). 

### 3.6. Influence of Hesperidin on Serum IgG, IgM and IgA Concentrations

**Figure 7:**
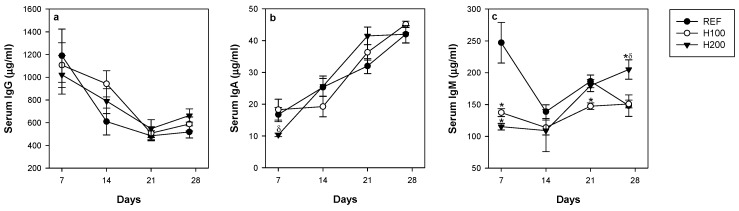
Effect of hesperidin administration throughout the study on serum (**a**) IgG; (**b**) IgA; and (**c**) IgM. Data are expressed as mean ± standard error (*n* = 5–6). Statistical difference: * *p* < 0.05 versus REF group, ^δ^ *p* < 0.05 versus H100 group (Mann–Whitney *U* test).

The influence of hesperidin on systemic immunity was assessed by determining the concentrations of serum IgG, IgA, and IgM throughout the study (Figure 7a–c). None of the hesperidin administrations altered the time-course of serum IgG (Figure 7a) and IgA concentrations (Figure 7b) in comparison to the REF group. On the contrary, one week of both doses of hesperidin was able to significantly reduce the amount of serum IgM (Figure 7c), but such effect was not observed over the entire study. In fact, and interestingly, serum IgM concentration was increased after four weeks of the highest dose of hesperidin (Figure 7c).

## 4. Discussion

Previous studies showed the effect of hesperidin administration on the intestinal immune system in infected mice [22] and in immunized rats [23]. Moreover, the influence of hesperetin, the hesperidin aglycone, on the gut microbiota has been reported [24]. However, as far as we know, the influence of hesperidin in the gut-associated lymphoid tissue on health status as well as its association with gut microbiota has not been previously described. Here we aimed to investigate in-depth the effects of hesperidin on the immune system, focusing on the gut-associated lymphoid tissue and the gut microbiota in healthy rats. 

The hesperidin doses used in this study (100 or 200 mg/kg) were established with the aim of observing the effects on both the intestinal immune system and on the microbiota, and taking into account that in a previous study a diet including hesperidin did not alter the gut microbiota composition in rats [24]. The dosage used (oral gavage, three times per week for four weeks) had already been used in a previous study, producing higher immunomodulatory effects than the incorporation of hesperidin in the rat food [23], and as observed here, it did not affect the food and water intake of the animals, nor did their body weight increase. The equivalence between rat and human dosage can be established by taking into account the body surface area [41]. Therefore, 100 mg/kg hesperidin in rat would be equal to 16.22 mg/kg hesperidin in humans, which would be supplied by more than a litre of orange juice for an adult (given that 1 L of orange juice provides about 600 mg hesperidin [42]). 

Interestingly, we found that hesperidin administration influenced the gut microbiota composition. The H200 group increased the total bacteria number in caecal content nearly twofold when compared to the reference group. In particular, the administration of 200 mg/kg hesperidin promoted the growth (total counts) of *Streptococcus* spp., *Lactobacillus/Enterococcus*, *Staphylococcus* spp., *Bacteroides/Prevotella*, *Bifidobacterium,* and *E. coli.* Furthermore, this high hesperidin dose did not affect the growth of *C. coccoides/E. rectale* and *C. histolyticum/C. perfringens* groups. In addition, considering their proportions, there was a higher percentage of *Lactobacillus* and *Staphylococcus* spp. Given that *Lactobacillus* genus is associated with many beneficial effects for the host, promoting anti-inflammatory [43,44], anti-viral [45], and anti-diabetic actions [46], our results suggest the potential prebiotic-like effects of hesperidin. In partial agreement with our results, a recent in vitro study demonstrated a rise in bacterial growth, particularly on *Lactobacillus rhamnosus* and also *Bifidobacterium longum* cultures, by the presence of 25 µg/mL of hesperidin and also with its aglycone form [47]. However, another in vitro study showed no impact of hesperidin on *Lactobacillus* spp. cultures, and even though its aglycone form, hesperetin, inhibited their growth [48]. Considering in vivo studies, our findings are in line with a study in which a hesperidin derivate (neohesperidin dihydrochalcone) together with saccharin increased the content of *Lactobacillus* in caecal samples from piglets [49]. Likewise, we found that hesperidin increased the proportion of *Staphylococcus* spp., which does not agree with in vitro studies using berry phenols [50] and in vivo studies carried out with cocoa flavonoids [30,51]. While *Staphylococcus* genus could include opportunistic species such as *S. aureus*, it is worthy to note that an increase in *Staphylococcus* spp. counts has been described in rats after prebiotic fibre-enriched diets [52]. In addition, hesperidin induced a lower proportion of *C. coccoides* group, which was also found in a mouse model treated with a probiotic strain of *Lactobacillus* [53]. On the other hand, Unno et al. [24] showed that the ingestion of a hesperetin-enriched diet for three weeks, but not one containing hesperidin, modified the microbiota composition in rats. The difference in the results between hesperetin and hesperidin was attributed to the higher inhibitory effect of hesperitin on starch digestion, increasing the short-chain fatty acid production in the large intestine. These authors postulated that the attachment of the rutinose moiety to the flavanone structure diminished its effectiveness [24]. On the other hand, the discrepancies with the reported and current results could be attributed to the dosage of the flavanone, which could influence its bioavailability, and which could also be affected by the food matrix. In addition, the different techniques used to assess microbiota composition, as well as the bacterial genera and species investigated cannot be discarded as factors influencing the outcomes. 

In relation to IgA, although no differences were found in faecal and serum IgA as a result of the nutritional intervention, it is worth noting that both doses of hesperidin were able to increase the IgA content in the small intestine. Interestingly, such an effect had already been observed with the administration of 100 mg/kg hesperidin, thus behaving differently than on the microbiota. As IgA is the main immunoglobulin in the mucosal surfaces providing a first line of non-inflammatory immune defense in the organism [54], our results suggest that hesperidin may play a principal role in the maintenance of the gut homeostasis by increasing intestinal IgA in healthy animals. In line with this, the ingestion of a 0.5% hesperidin-enriched diet increased small intestine IgA levels in orally sensitized rats [23], as did other dietary polyphenols such as curcumin and those extracted from Japanese fruits [55,56]. On the other hand, no differences were observed, quantifying caecal and faecal IgA, suggesting that the rise in the small intestine IgA content was coated by the higher number of caecal bacteria, as shown by the increase in the number of caecal IgA-coated bacteria. In any case, a higher amount of small intestine IgA and bacteria coated to IgA, as well as beneficial changes in the caecal microbiota, will enhance the intestinal barrier to protect against bacterial invasion, and to compete with pathogenic microorganisms for enterocyte adherence.

Regarding the hesperidin mechanism involved in increasing small intestine IgA content, the study of the gene expression of molecules implicated in the proliferation, differentiation, and migration of IgA+ B cells was inconsistent. Other mechanisms, such as IgA transcytosis and also T cell-independent mechanisms associated with TLR expression, deserve further attention. While the intestinal cytokines were not useful in elucidating the implicated mechanism, we found a decrease in the pro-inflammatory cytokines IFN-γ and MCP-1, which agrees with the anti-inflammatory properties of hesperidin reported in vitro [57] and in a model of neuroinflammation [14]. These downregulatory effects on pro-inflammatory cytokines could be useful even in health status, as in the current study, avoiding lower-grade inflammation due to innocuous antigens and promoting tolerance. The effects on IFN-γ and MCP-1 do not agree with a previous study carried out in rats immunized with ovalbumin and adjuvants challenging Th2-response [23], indicating the different responses of the immune system when it was specifically triggered to be polarized for a particular immune response. Nonetheless, modifications in the gut associated lymphoid tissue (GALT) were detected through changes in MLN. In this sense, a difference in the T and B lymphocyte proportions were observed, with a higher T cell percentage, which positively correlates with the increase in small intestine IgA levels, showing a major sensibility in the lowest dose of hesperidin. These changes in the T and B cell proportions did not impact on their functionality assessed by the proliferation test and the cytokine secretion. Therefore, further studies should be focused on the hesperidin mechanism of action on the GALT, which finally enhances small intestine IgA content. The effects of hesperidin on the intestinal IgA are not reflected at systemic level. Serum IgG and IgA concentrations were not modified by the flavanone, and only punctual effects on serum IgM levels were observed. In fact, effects on serum immunoglobulin or specific antibody concentrations by hesperidin have not been reported before [23], with the exception of an attenuating action on ovalbumin-specific serum levels of IgE in an asthma mouse model [58].

## 5. Conclusions

In conclusion, our study demonstrates that the administration of hesperidin (200 mg/kg, three times/week for four weeks) is able to influence gut microbiota by increasing the total bacteria number due to, among others, the increased proportions of *Lactobacillus* and *Bifidobacterium* genera, which suggests its potential prebiotic effect. Even though hesperidin showed no effect on serum IgG, IgA, and IgA concentrations, it could play a key role in the gut homeostasis maintenance by increasing intestinal IgA at a lower dose than that required to induce the prebiotic effect. These effects were observed in healthy animals and suggest the potential of hesperidin in preventing and treating some immune-mediated diseases.

## Figures and Tables

**Table 1:** Oligonucleotide probes and hybridization conditions used in the analysis of intestinal bacteria by FISH-FCM. Y = (C/T), R = (A/G).

Phylum	Bacterial Group Targeted	Sequence (5′–3′)	Hybridization Temperature (°C)	Reference
*Firmicutes*	*Clostridium coccoides/Eubacterium rectale*	GCTTCTTAGTCARGRACCG	50	[32]
	*Clostridium histolyticum/C. perfringens*	TTATGCGGTATTAATCT(C/T)CCTTT	50	[33]
	*Lactobacillus/Enterococcus*	GGTATTAGCAYCTGTTTCCA	50 (lysozyme)	[34]
	*Staphylococcus* spp.	TCCTCCATATCTCTGCGC	46	[35]
	*Streptococcus* spp.	CACTCTCCCCTTCTGCAC	46	[35]
*Bacteroidetes*	*Bacteroides/Prevotella*	CCAATGTGGGGGACCTT	46	[36]
*Actinobacteria*	*Bifidobacterium* spp.	CATCCGGCATTACCACCC	50	[37]
*Proteobacteria*	*Escherichia coli*	CACCGTAGTGCCTCGTCATCA	37	[38]

**Table 2:** Effect of hesperidin administration on body weight, food and water intake throughout the study. Body weight is expressed as mean ± standard error (*n* = 6). Food and water intake data are expressed as mean ± standard error obtained from two cages (3 rats/cage).

Day	Body Weight (g)			Food Intake (g/100 g rat/day)			Water Intake (g/100 g rat/day)		
	REF	H100	H200	REF	H100	H200	REF	H100	H200
**0**	48.8 ± 1.6	48.5 ± 0.8	48.4 ± 0.9	-	-	-	-	-	-
**7**	77.2 ± 2.6	75.3 ± 1.4	74.8 ± 1.4	14.3 ± 0.5	14.7 ± 0.1	14.7 ± 0.2	10.8 ± 0.1	11.1 ± 0.2	13.8 ± 2.5
**14**	117.0 ± 3.8	117.6 ± 1.9	119.2 ± 2.2	11.80± 2.4	9.2 ± 0.0	10.0 ± 0.8	10.9 ± 0.0	12.0 ± 0.2	11.6 ± 0.3
**21**	158.0 ± 3.8	162.6 ± 1.8	161.1 ± 3.0	12.6 ± 0.2	12.7 ± 0.4	12.3 ± 0.2	10.1 ± 0.3	11.6 ± 0.3	10.7 ± 0.1
**28**	190.5 ± 4.7	189.9 ± 1.9	189.9 ± 3.3	9.8 ± 0.3	9.8 ± 0.0	9.4 ± 0.2	11.5 ± 0.0	11.7 ± 0.2	11.0 ± 0.8

**Table 3:** Effect of hesperidin administration on gene expression of some molecules in small intestine. The relative mRNA gene expression was calculated assigning the value of 100% to the mean of the rats from the REF group. Data are expressed as mean ± standard error (*n* = 5–6).

Gene	Relative Gene Expression (%)		
	REF	H100	H200
**IgA**	100.00 ± 13.24	148.46 ± 82.70	151.91 ± 37.14
**TGF-β**	100.00 ± 11.88	63.00 ± 14.39	85.96 ± 23.61
**CCR9**	100.00 ± 12.94	69.05 ± 14.85	113.66 ± 31.96
**CD40**	100.00 ± 36.80	82.97 ± 30.91	62.36 ± 12.78
**CCL25**	100.00 ± 13.80	66.57 ± 22.26	102.75 ± 17.79
**CCL28**	100.00 ± 15.62	104.00 ± 14.08	100.00 ± 14.14

## Supplementary Materials

The following are available online at http://www.mdpi.com/2072-6643/11/2/324/s1, Figure S1: Effect of hesperidin administration on counts of each bacterial group determined by FISH-FCM in caecal homogenates, Table S1: Composition of the diet (Teklad Global 14% Protein Rodent Maintenance Diet, Madison, USA).
