# Supplementary material for: Hesperidin Effects on Gut Microbiota and Gut-Associated Lymphoid Tissue in Healthy Rats
Source: Nutrients. 2019 Feb 2;11(2):324. doi: 10.3390/nu11020324 (PMC6412496; doi:10.3390/nu11020324)
Supplement: Supplementary file 1 [file nutrients-11-00324-s001.pdf]

Table S1. Composition of the diet (Teklad Global 14% Protein Rodent Maintenance Diet, Madison, USA).

**Components**	**%**
Crude Protein	14.3
Fat (ether extract)	4.0
Carbohydrate (available)	48.0
Crude Fibre	4.1
Neutral Detergent Fibre	18.0
Minerals	2.8
Humidity	8.8

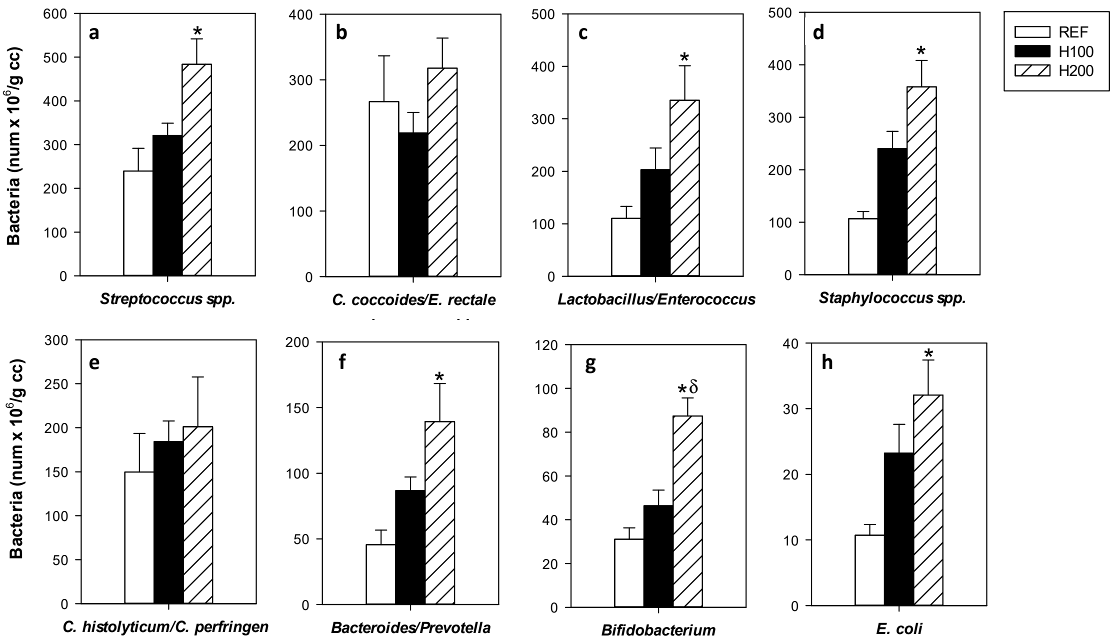

Figure S1. Effect of hesperidin administration on counts of each bacterial group determined by FISH-FCM in caecal homogenates. (**a**) *Streptococcus spp.*; (**b**) *Clostridium coccoides/Eubacterium rectale*; (**c**) *Lactobacillus/Enterococcus*; (**d**) *Staphylococcus spp.*; (**e**) *Clostridium histolyticum/Clostridium perfringens*; (**f**) *Bacteroides/Prevotella*; (**g**) *Bifidobacterium*; and (**h**) *Escherichia coli*. Data are expressed as mean ± standard error (n = 5−6). Statistical difference: * $p < 0.05$ versus REF group, δ $p < 0.05$ versus H100 group (one-way ANOVA)
